# AMBER: advancing multimodal brain-computer interfaces for enhanced robustness—A dataset for naturalistic settings

**DOI:** 10.3389/fnrgo.2023.1216440

**Published:** 2023-08-24

**Authors:** Muhammad Ahsan Awais, Peter Redmond, Tomas Emmanuel Ward, Graham Healy

**Affiliations:** Insight Science Foundation Ireland Research Centre for Data Analytics, School of Computing, Dublin City University, Dublin, Ireland

**Keywords:** EEG, BCI, artifacts, signal denoising, P300 ERPs, RSVP, noise, EEG dataset

## 1. Introduction

The AMBER dataset aims to support researchers in developing signal-denoising techniques that mitigate the impact of noise sources such as eye blinks, eye gaze, talking and body movements, in order to ameliorate the signal-to-noise characteristics of EEG (Electroencephalography) measurements. The emphasis of this research is to enable the robust performance of brain-computer interface systems in naturalistic real-world settings, i.e., outside of the lab.

Prior studies (Manor et al., 2016; Huang et al., 2020; Ranjan et al., 2021) have typically focused on using characteristics of EEG signals in isolation without contextual signal sources to identify and ameliorate such artifacts with limited success. Presently, however, no suitable dataset exists in order to train and evaluate such approaches.

Several research articles have discussed P300 RSVP datasets available to researchers (Lees et al., 2018). Notably, Won et al. (2022) collected P300 RSVP data from 50 participants using a 32-channel Biosemi ActiveTwo system, while another dataset was presented by Acqualagna and Blankertz (2013), acquired from 12 subjects. Additionally, an extensive dataset was introduced by Zhang et al. (2020), which involved 64 subjects recorded with a 64-channel Synamps2 system. However, these datasets share a common limitation in that they solely focus on EEG signals without incorporating any contextual signal sources. In contrast, our aim is to provide the research community with an extensive dataset that not only includes participant video data alongside EEG recordings, but also closely replicates real-world situations, bridging the gap between controlled laboratory settings and naturalistic environments.

The AMBER dataset incorporates a multimodal and contextual approach by capturing video data of the participant alongside the EEG recording. The inclusion of video data enriches the dataset by providing contextual information and additional modalities for analysis. By recording video data simultaneously with EEG signals, researchers can leverage a broader range of contextual cues, such as facial expressions, body movements, and eye movements.

EEG is an accessible and safe method for researchers and users to build and operate BCI (Brain-Computer Interface) applications (Awais et al., 2021). While the initial use of such techniques began in clinical/rehabilitative settings for the purposes of augmenting communication and control, a recent trend has been to use such signals and methods in new domains, such as the image annotation task, which relies on the identification of target brain events to trigger labeling (Bigdely-Shamlo et al., 2008; Pohlmeyer et al., 2011; Marathe et al., 2015).

The Rapid Serial Visual Presentation (RSVP) is an approach to BCI in which a series of images are displayed at high speed. Participants are asked to differentiate between a set of target images and a set of standard images, where P300 ERP is evoked by a target image but not by standard images (Healy and Smeaton, 2017; Wang et al., 2018).

While the RSVP-BCI paradigm can be demonstrated in controlled lab-based environments, translation of this paradigm into consumer contexts requires a better understanding of real-world EEG artifacts that impact EEG signal quality in ecologically valid settings, e.g., in online worlds, metaverse, and gaming contexts. Such application scenarios are characterized by less constrained user behavior, some of which is entirely necessary for the normally expected interactions. Examples include talking, head and hand movements, all of which generate artifacts that impede the application of EEG in BCIs in real-world contexts.

By utilizing the P300/RSVP task in this dataset, we aim to differentiate the EEG results obtained in the presence of noise from those obtained in noise-free conditions. This differentiation allows for a comprehensive analysis of the impact of noise on EEG signals and facilitates the evaluation of signal-denoising techniques. The P300/RSVP task is particularly relevant as it involves measuring accuracy, making it an effective dependent variable to assess the efficacy of noise cleaning and evaluate the influence of behavioral artifacts on the EEG signals. Furthermore, we can gain insights into the relationship between noise, behavioral artifacts, and the quality of EEG signals, ultimately enhancing our understanding of the robustness of EEG data in real-world settings.

For the purpose of creating this dataset, participants were instructed to produce particular artifacts at particular times via a carefully controlled protocol, e.g., moving head left to right vs. up and down, eye movement, eye blinks, facial expressions, lip movement, body movement, etc. The specific artifacts that participants were instructed to produce during data recording reflect the most problematic artifacts encountered in real-world EEG recording (Urigüen and Garcia-Zapirain, 2015; Jiang et al., 2019; Rashmi and Shantala, 2022).

Moreover, the AMBER dataset represents a significant advancement in the field of brain-computer interfaces (BCIs) by providing researchers with a resource to address one of the key challenges in EEG data analysis—signal denoising. EEG recordings are often plagued by various artifacts and noise, which can obscure the underlying neural signals and hinder accurate analysis. The importance of signal-denoising lies in its potential to enhance the quality and reliability of EEG measurements, enabling more accurate identification and interpretation of neural responses. By effectively removing unwanted artifacts and noise, researchers can gain deeper insights into brain activity and cognitive processes, leading to a more comprehensive understanding of neural mechanisms. Moreover, denoising techniques are crucial for developing robust BCIs that can reliably detect and interpret brain signals in real-world scenarios. The dataset serves as a valuable resource for exploring and developing novel signal denoising techniques, ultimately paving the way for more effective and practical applications of BCIs in naturalistic settings.

## 2. Data acquisition

Ten healthy participants aged between 20 and 35 years were recruited from Dublin City University to participate in the data collection. Among these participants, there were 6 males and 4 females, and each participant was assigned an alias ranging from “P1” to “P10”. Data acquisition was performed with approval from the Dublin City University (DCU) Research Ethics Committee (DCUREC/2021/175).

The hardware used for data collection was the ANT-Neuro eego sports mobile EEG system. A 32-channel EEG cap positioned according to the 10-20 international electrode system was used for the data acquisition. CPz was used as the online reference channel, and the impedance of all electrodes was kept under 15 kOhm. Data was collected at a sampling rate of 1,000 Hz (using a lowpass filter of 500 Hz) and saved in EDF format.

EEG was recorded from the ten participants while they followed a pre-defined protocol of tasks. Timestamp information for image presentation (via a photodiode and hardware trigger) was also captured to allow for precise epoching of the EEG signals for each trial (Wang et al., 2016).

## 3. Experimental protocol

In the AMBER dataset, we employ a multimodal approach that encompasses two primary signal sources: (1) EEG data collection and (2) video recording. Both EEG recordings and video are captured at the same time.

### 3.1. EEG recording

The dataset contains EEG responses to 10,500 images, in total. Each participant completed 4 sessions where each session contained 8 blocks followed by 3 different baselines. A description of each task in a single session is given in Table 1.

**Table 1:** Description of tasks performed in a single session.

**No**.	**Task ID**	**Task description**	**Duration (s)**
1.	B1	Baseline Eyes Open	60
2.	B0	Baseline Eyes Movement	10
3.	B2	Baseline Eyes Close	60
4.	X1	RSVP Task	90
5.	X2	RSVP Task	90
6.	X3	Artifact 1: Body Movement	90
7.	X4	RSVP + Body Movement	90
8.	X5	Artifact 2: Talking	90
9.	X6	RSVP + Talking	90
10.	X7	Artifact 3: Head Movement	90
11.	X8	RSVP + Head Movement	90

As seen in Table 1, the data collection is split into three sections:

1. The standard RSVP image search paradigm, in which the subjects perform the target search task while sitting still in front of the monitor (X1 and X2);
2. The RSVP paradigm with participant-induced noise, in which the participants through following a protocol, generate three distinct types of EEG artifactual noise through movements (i.e., head movement, body movement, and talking) while doing the RSVP task (X4, X6, and X8);
3. The noise paradigm, in which the participants generate three distinct types of noises throughout the trial without performing any RSVP image search task (X3, X5, and X7).

**Figure 1:**
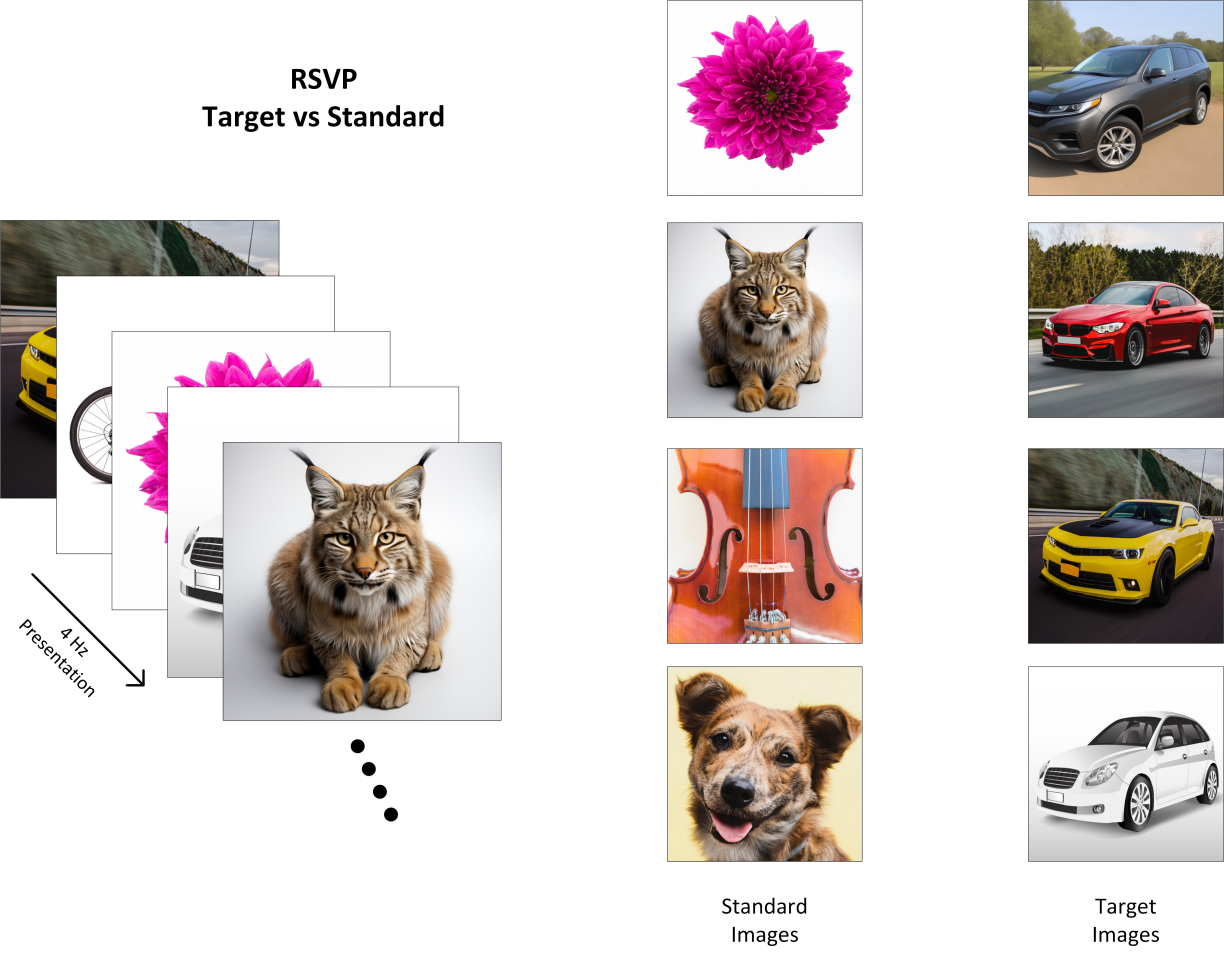
Illustration of the P300/RSVP task where images of cars have been used as the target class. Reproduced with permission from Freepik.com^1^.

In the image search RSVP task, participants searched for a known type of target (e.g., a car) and were instructed to covertly count occurrences of target images in the RSVP sequence so as to maintain their attention on the task. In Figure 1, we show examples of the target search images used. In each 90-s RSVP block, images were presented successively at a rate of 4 Hz with target images randomly interspersed among standard images with a percentage of 10% across all blocks. In each block, 360 images (36 targets/324 standards) were presented in rapid succession on screen.

There were 288/2592 target/standard trials captured for the standard RSVP task per participant, whereas, in the case of each noisy RSVP task (1-body movement, 2-talking, and 3-head movement), there were 144/1296 target/standard trials available.

In addition to recording using the pre-defined RSVP paradigm, intentional artifacts were also generated by the participants through following a protocol. In the first scenario, participants generated noise in parallel with the RSVP task, and in the second scenario, they generated artifactual noise without performing the RSVP task.

These intentional artifacts were induced to simulate realistic scenarios and study the effects of body movement, talking, and head movement on EEG data. By performing these intentional artifact-generating tasks in parallel with the RSVP task, this dataset captures the effect of coincident noise on task performance and EEG data. The inclusion of intentionally generated artifacts also enables a more comprehensive analysis of the noise characteristics and their impact on EEG data, enabling researchers to develop more effective signal-denoising techniques.

#### 3.1.1. Artifact 1: body movement

To induce body movement artifacts, participants were instructed to repeatedly raise and wave their hands, followed by putting their hands down, and repeating this sequence of movements for the entire duration of the 90-s block. Participants were given the freedom to choose which hand(s) to raise and in what sequence, with the intention of inducing variability around artifact production. They were instructed to perform the task at a comfortable speed, neither too fast nor too slow, in order to maintain consistency across participants. This task was designed to simulate a real-world or metaverse-type environment where a person might be moving their hands and arms while using a virtual reality setup such as playing a game or engaging in any other activity that involves body movements.

#### 3.1.2. Artifact 2: talking

To generate talking artifacts, participants were instructed to count aloud, mixing numbers and letters in random sequences, for the entire 90-s block. They were given the freedom to choose the order and sequence of their counting, with the aim of inducing variability.

During the RSVP task, participants were instructed to count and continuously repeat the number of target images out loud to create talking artifactual noise in the EEG. The aim of this instruction was to simulate a real-life scenario where individuals may need to focus on a task while simultaneously communicating verbally in situations like playing a game or collaborating with others in a virtual space.

#### 3.1.3. Artifact 3: head movement

To generate head movement artifacts, participants were instructed to move their heads in an up-down (nodding) or left-right motion, at a natural speed, throughout the entire 90-s block. Participants were specifically instructed to alternate between up-down and left-right head movements in a randomized manner. This task was designed to simulate real-world scenarios where a person might produce head movements in situations like nodding in agreement during a conversation or shaking their head in response to a question. Additionally, this task aimed to simulate virtual reality (VR) environments, where a person might use a VR headset and move their head to explore different directions while playing games or visiting virtual spaces.

**Figure 2:**
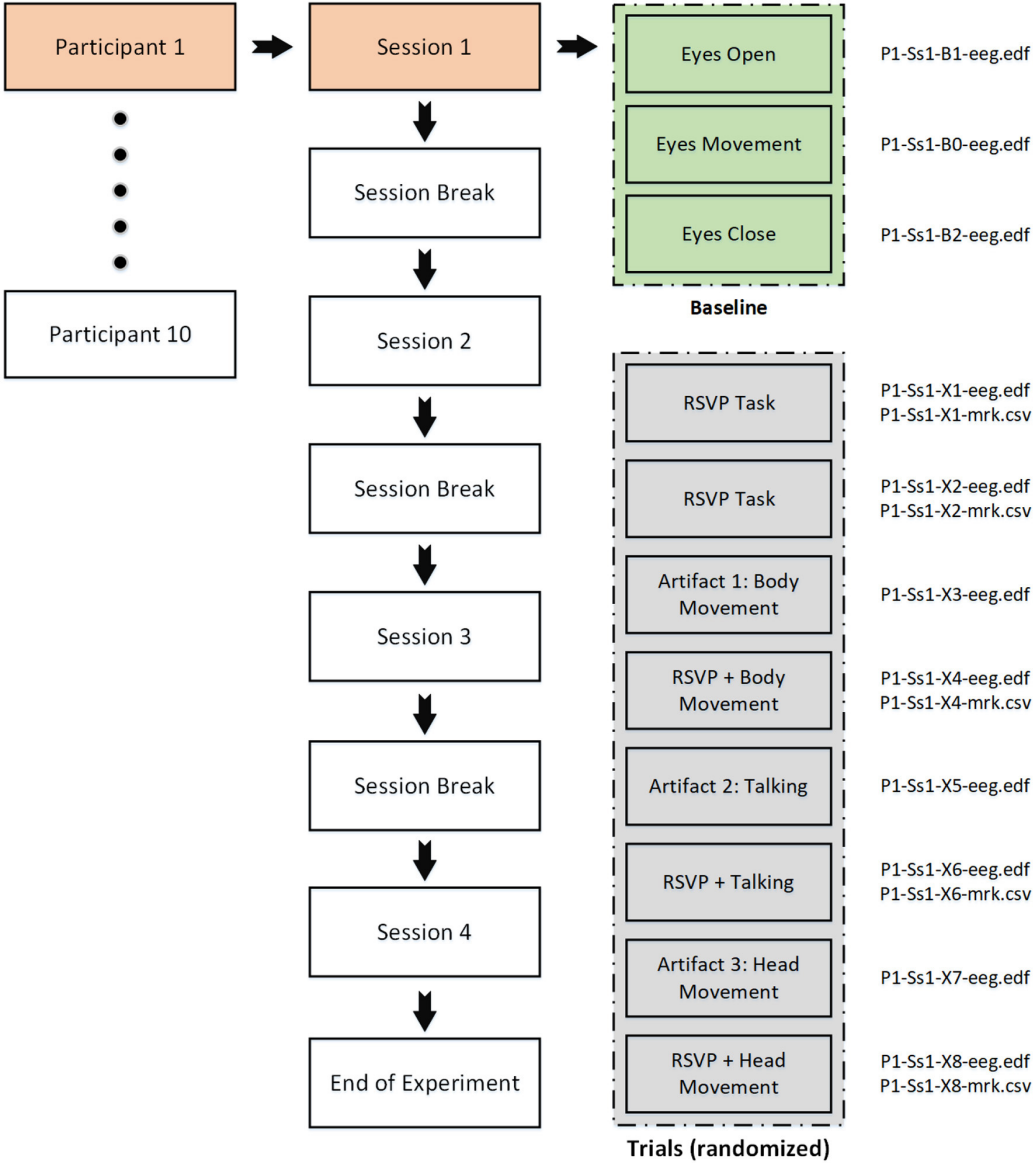
Organization of the EEG recordings for each participant.

Figure 2 illustrates the organization of the EEG recordings for each participant. It also provides an overview of the files (i.e., raw EEG data and labels) associated with each task.

### 3.2. Video recording

We employed a multimodal approach that involved capturing video data of BCI participants while they produced specific artifacts.

#### 3.2.1. Video data collection and framing

To accurately capture the various artifacts, video data was recorded using a Logitech C920 webcam, which has a frame rate of 20 fps. The camera was calibrated using OpenCV (Zhang, 2000) to determine the camera matrix and distortion coefficients, ensuring the accuracy of the recorded video data.

During the recording of eye, head, and mouth-related artifacts, the participant's face was kept fully in the frame. This framing approach allowed for a clear and unobstructed view of the participant's movements, including pupil dilation, eye-opening size, and movement. For arm movements, the upper torso was maintained in full frame, ensuring proper visibility of the body movements.

#### 3.2.2. Video data annotation and time-stamping

Each video frame was timestamped and assigned a corresponding frame number, which was recorded in a corresponding CSV file. This file also contained information on frame size, zoom factors, and camera matrix. The timestamping process ensured that the video data could be accurately synchronized with the EEG data, allowing for a robust multimodal analysis.

#### 3.2.3. Significance of video data in contextual EEG dataset

The integration of video data into the Contextual EEG Dataset is critical for developing advanced signal-denoising techniques and improving BCI performance in real-world settings. Combining video data with electroencephalogram (EEG) recordings enables researchers to explore the correlations between facial expressions, head movements, and brain activity, leading to a better understanding of various (neuro-)physiological phenomena. By capturing these problematic artifacts encountered in naturalistic environments, researchers can better understand the factors affecting EEG data quality and develop solutions to mitigate their impact on BCI performance.

## 4. Data records

In this work, we collected EEG data from 10 participants, which is labeled as Dataset A. For illustrative purposes, we conducted basic pre-processing steps on the raw EEG data, resulting in a modified version referred to as Dataset B. Alongside the EEG recordings, we simultaneously captured video data, which was independently processed to extract relevant information. These video-derived extractions are denoted as Dataset C. The availability of multiple datasets enables comprehensive analysis, allowing us to explore the relationship between EEG signals, video data, and their potential combined insights. Detailed descriptions of each dataset are provided below.

### 4.1. Data A: raw EEG data

The raw EEG data recorded during each task is stored in EDF file format, which contains all the relevant information, including the complete EEG signal as well as the events that occurred during the task. For each participant, there are 11 EDF files corresponding to each session, which results in a total of 44 files for all four sessions.

The RSVP tasks have additional information about the target and standard image triggers, which are given in the form of CSV files. There are five RSVP tasks per session (two standard and three noisy RSVP tasks), which results in 20 CSV marker files for each participant. This combination of EDF and CSV files provides a comprehensive dataset that allows for detailed analysis of EEG signals and their corresponding events during the RSVP tasks. An overview of the files (i.e., EDF and CSV) associated with each task in a session can be seen in Figure 2.

During each RSVP task, a total of 360 images were presented to participants. As a result, each corresponding CSV marker file contains 360 rows of information having two distinct labels, “1” and “2”. The label “1” indicates the presence of a target image in the corresponding epoch for the RSVP task, while the label “2” indicates the absence of a target image, and, therefore, a standard non-target image. These labels provide vital information for the analysis of the EEG data as they allow the identification of the specific moments in time when the target and non-target stimuli were presented during the task.

### 4.2. Data B: processed EEG data

In addition to the raw data (i.e., Dataset A), an illustrative pipeline has been provided that encompasses crucial preprocessing steps to make the EEG data amenable to analysis, visualization, and machine learning. This transformation procedure was proposed as an example to restructure the continuous raw data into a more compact dataset and to make it easier to use.

The pipeline includes essential stages such as filtering, resampling, re-referencing, and epoching. By following this example pipeline, researchers can effectively transform and structure the data, allowing for a deeper understanding of its characteristics and facilitating further analysis. Python (version 3.9.12) was mainly used along with the MNE (version 0.24.0) to implement the processing. This example pipeline is available (**click here**), allowing interested researchers to modify the processing setup as they wish.

#### 4.2.1. Raw data loading

A function was developed in Python to quickly load the raw EEG data for a particular participant and session. This function is helpful for efficiently accessing the EEG data for analysis and processing. The raw data is stored in the EDF file format, which contains all the relevant information related to the EEG recordings, including the event markers. Further information about event markers is stored in separate CSV files for each RSVP task.

#### 4.2.2. Digital filtering

A low pass filter with a cut-off frequency of 30 Hz was applied to the EEG data to remove any high-frequency noise or artifacts that may be present in the signal above 30 Hz. This filtering step helped to improve the signal-to-noise ratio and enhance the quality of the EEG data.

#### 4.2.3. Re-sampling

We have carried out down-sampling on our original signal sampled at 1,000 Hz to obtain a down-sampled signal with a new sampling rate of 100 Hz. The down-sampled signal can help reduce the computational load of subsequent processing steps while preserving the essential features of the signal.

#### 4.2.4. Re-referencing

The raw EEG data was initially recorded using the default CPz reference. However, in order to improve the quality of the data, common average referencing (CAR) was applied using the MNE re-reference function.

#### 4.2.5. Epoching

The continuous EEG recordings were segmented into epochs by extracting the time series data from −0.2 to 1 s relative to the onset of the visual stimulus.

After undergoing the aforementioned pre-processing steps, the data is saved into CSV files. Each session comprises 11 CSV files, resulting in a total of 44 files for each participant. Each file contains comprehensive information about the channels, epochs, and their respective categories. By providing access to this pre-processed data as an example, users can explore the raw datasets in a multitude of ways, unlocking various possibilities for analysis and interpretation.

### 4.3. Data C: video data

In this section, we describe the process of extracting metadata from video recordings using OpenCV libraries and synchronizing it with EEG data recorded in the European Data Format (EDF).

#### 4.3.1. Video processing and metadata extraction

Video recordings can provide valuable information on an individual's facial expressions, head position, and skeletal movements. To extract this information, we utilized the OpenCV libraries, which offer a wide range of functionalities for image and video analysis. The metadata extracted includes:

1. Three-dimensional head position.
2. Eye opening.
3. Mouth opening.
4. Two-dimensional eye position.
5. Three-dimensional skeleton movement.

Each of these metadata elements is timestamped to ensure accurate synchronization with the EEG data.

#### 4.3.2. Data conversion and representation

Once the metadata is extracted from the video, it is converted into a suitable format for integration with the EEG data. We represent each metadata element as a graph line to facilitate the visualization and analysis of the combined data. This graphical representation allows researchers to easily identify patterns and correlations between video metadata and EEG activity.

#### 4.3.3. Synchronization with EEG data

In order to accurately integrate the video metadata with the EEG data, we utilize the video frame timestamps as a reference for aligning the metadata with the EEG data. This ensures that each metadata element is correctly associated with the corresponding EEG activity.

The combined analysis of video metadata and EEG data has numerous applications in various fields, such as neuroscience, psychology, and human-computer interaction. By understanding the relationship between facial expressions, head movements, and brain activity, researchers can gain insights into emotion recognition, attention, and cognitive processes. Moreover, this approach can also be applied to develop advanced human-computer interfaces and improve the accuracy of brain-computer interfaces (Redmond et al., 2022).

## Data availability statement

The datasets presented in this study can be found in online repositories. The names of the repository/repositories and accession number(s) can be found at: https://github.com/meharahsanawais/AMBER-EEG-Dataset.

## Ethics statement

The studies involving humans were approved by Dublin City University (DCU) Research Ethics Committee (DCUREC/2021/175). The studies were conducted in accordance with the local legislation and institutional requirements. The participants provided their written informed consent to participate in this study.

## Author contributions

MA, PR, GH, and TW have made substantial contributions to this research work. MA designed the EEG-based experimental paradigm and conducted the data acquisition. PR contributed by designing the video recording protocols. GH contributed by designing the RSVP task and reviewing the paper. TW reviewed and finalized the manuscript for publication. All authors contributed to the article and approved the submitted version.

## Footnotes

^1^Freepik.com image source links: https://www.freepik.com/free-photo/red-luxury-sedan-road_6198664.htm#query=cars&position=0&from_view=search&track=sph; https://www.freepik.com/free-photo/yellow-sport-car-with-black-autotuning-road_6159512.htm#query=cars&position=2&from_view=search&track=sph#position=2&query=cars; https://www.freepik.com/free-vector/white-hatchback-car-isolated-white-vector_3602751.htm#from_view=detail_alsolike; https://www.freepik.com/free-photo/pink-flower-white-background_976070.htm#query=flower&position=5&from_view=search&track=sph; https://www.freepik.com/free-photo/close-up-fine-tuning-string-adjuster-violin_2978632.htm#query=wooden%20guitar%20on%20table&position=27&from_view=search&track=ais; https://www.freepik.com/free-photo/front-view-cute-domestic-dog-pet_5795881.htm#query=golden%20retriever%20face&position=1&from_view=search&track=ais; https://www.freepik.com/free-ai-image/scary-bobcat-indoors_59584025.htm#query=bengal%20cat%20close%20up&position=3&from_view=search&track=ais; https://www.freepik.com/free-ai-image/gray-suv-is-parked-road-front-field_40368536.htm#query=cars&position=36&from_view=search&track=sph.
